# Targeting the hSSB1-INTS3 Interface: A Computational Screening Driven Approach to Identify Potential Modulators

**DOI:** 10.1021/acsomega.3c09267

**Published:** 2024-02-08

**Authors:** Tabassum Khair Barbhuiya, Sam Beard, Esha T. Shah, Steven Mason, Emma Bolderson, Ken O’Byrne, Luke W. Guddat, Derek J. Richard, Mark N. Adams, Neha S. Gandhi

**Affiliations:** † Centre for Genomics and Personalised Health, and School of Chemistry and Physics, Faculty of Science, Queensland University of Technology, 2 George Street, Brisbane, QLD 4000, Australia; ‡ Cancer and Ageing Research Program, Woolloongabba, QLD 4102, Australia; § Centre for Genomics and Personalised Health, and School of Biomedical Sciences, Faculty of Health, Queensland University of Technology, Kelvin Grove, QLD 4059, Australia; ∥ School of Chemistry and Molecular Biosciences, The University of Queensland, Brisbane, QLD 4072, Australia

## Abstract

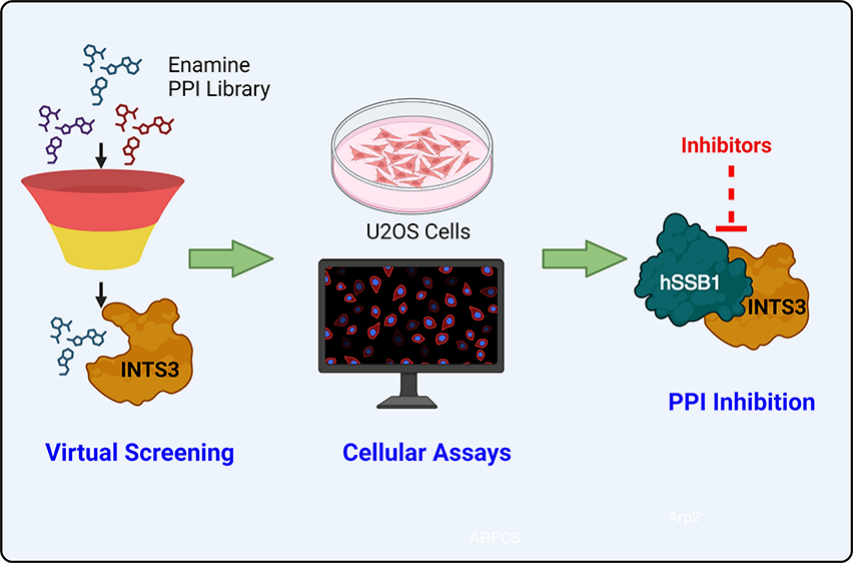

Human single-stranded DNA binding protein 1 (hSSB1) forms a heterotrimeric complex, known as a sensor of single-stranded DNA binding protein 1 (SOSS1), in conjunction with integrator complex subunit 3 (INTS3) and C9ORF80. This sensory protein plays an important role in homologous recombination repair of double-strand breaks in DNA to efficiently recruit other repair proteins at the damaged sites. Previous studies have identified elevated hSSB1-mediated DNA repair activities in various cancers, highlighting its potential as an anticancer target. While prior efforts have focused on inhibiting hSSB1 by targeting its DNA binding domain, this study seeks to explore the inhibition of the hSSB1 function by disrupting its interaction with the key partner protein INTS3 in the SOSS1 complex. The investigative strategy entails a molecular docking-based screening of a specific compound library against the three-dimensional structure of INTS3 at the hSSB1 binding interface. Subsequent assessments involve *in vitro* analyses of protein–protein interaction (PPI) disruption and cellular effects through co-immunoprecipitation and immunofluorescence assays, respectively. Moreover, the study includes an evaluation of the structural stability of ligands at the INTS3 hot-spot site using molecular dynamics simulations. The results indicate a potential *in vitro* disruption of the INTS3-hSSB1 interaction by three of the tested compounds obtained from the virtual screening with one impacting the recruitment of hSSB1 and INTS3 to chromatin following DNA damage. To our knowledge, our results identify the first set of drug-like compounds that functionally target INTS3-hSSB1 interaction, and this provides the basis for further biophysical investigations that should help to speed up PPI inhibitor discovery.

## Introduction

DNA double-strand breaks (DSBs) are one of the most cytotoxic lesions caused by endogenous damaging events such as replication stress, reactive oxygen species, and exogenous agents such as ionizing radiation (IR) and chemical agents.^1−3^ If not repaired or improperly repaired, the damaged DNA can cause abnormalities in cellular functions and disease progression.^4,5^ The DSBs occurring in the G2 phase of the cell cycle are mainly repaired by the error-free, homologous recombination (HR)-mediated repair pathway.^6,7^ The HR repair is initiated with 3′-end resection, generating single-stranded DNA (ssDNA) overhangs.^8^ In eukaryotic cells, the ssDNA binding protein (SSB) complexes, e.g., RPA, the sensor of single-stranded DNA complex 1 and 2 (SOSS1 and SOSS2), bind to ssDNA and participate in DNA replication, repair, and recombination processes.^9−11^ Compared to RPA, the SOSS1 complex has a lower affinity for ssDNA binding.^12^ The heterotrimeric protein constituents of the SOSS1 complex, INTS3, hSSB1, and C9ORF80, have distinct roles in the DNA damage repair pathway. Depletion of either of the protein partners causes increased sensitivity of the cells to IR, defects in the cell cycle G2/M checkpoint, and impairment of HR-mediated repair.^11,13−16^ While hSSB1 is essential for the ssDNA binding activity of the SOSS1 complex, INTS3 acts as a key adaptor of the complex and is essential to its structural stability. It also has a functional role in mediating the accumulation of the SOSS1 complex at the DNA damage sites.^11,15,16^

**Figure 1:**
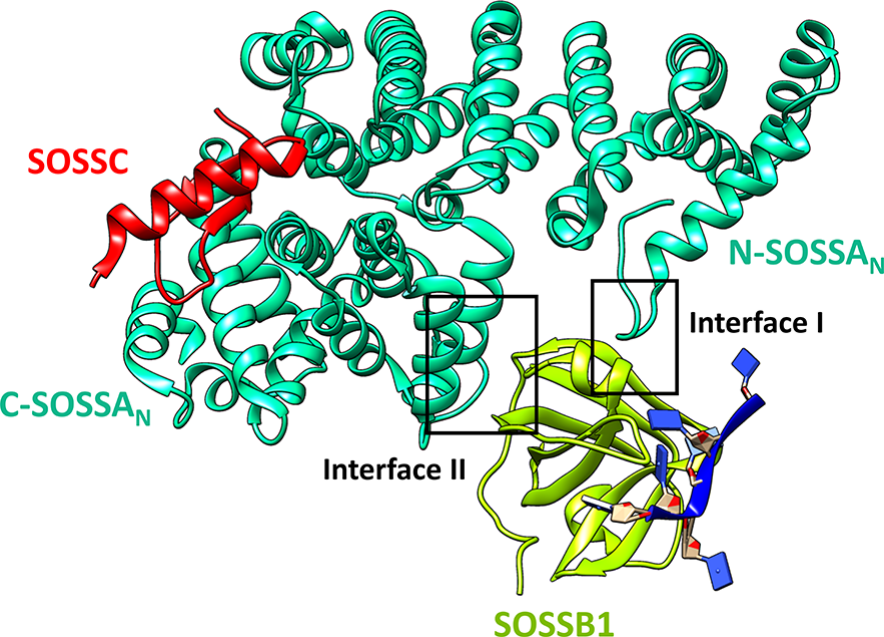
Crystal structure of the SOSS1 complex bound to ssDNA. The crystal structure of SOSS1 deposited in the Protein Data Bank (PDB ID: 4OWW) shows a ternary protein complex wherein 6-nucleotide ssDNA (dT6, represented in blue) binds to the OB-fold of SOSSB1 (hSSB1) at a site distinct from the SOSSA (INTS3) binding interfaces. The PPI interfaces of INTS3, interface I and II, bear loop and helical structures, respectively, at the N-terminus of INTS3, separating it from the SOSSC binding interface.

The crystal structure of the SOSS1 complex (PDB ID: 4OWW) with six nucleotides (6 nt) visible in the electron density map, poly-deoxythymidine (poly(dT)) represented in Figure 1, consists of three structural subunits: INTS3 designated as SOSSA (1–500 residues), hSSB1 or SOSSB1 (1–211 residues), and C9ORF80 or SOSSC (1–104 residues).^17^ The N-terminus of INTS3 (SOSSA_N_) consists of two structural domains, N-SOSSA_N_ (residues 32–292) and C-SOSSA_N_ (residues 306–498), acting as a bridge between SOSSB1 and SOSSC. In contrast, the C-terminus of INTS3 serves as a site for nucleic acid binding. The N- and C-domains of SOSSA_N_ are held together by an α-helical linker region, forming a 20 Å deep C-shaped cavity. INTS3 binds to the oligonucleotide binding (OB)-fold of hSSB1 at its protein–protein interaction (PPI) site, distinct from that of the oligonucleotide binding site. This PPI site consists of two interfaces: interfaces I and II, buried with a solvent-accessible surface area (SASA) of 1740 Å^2^.^17^ Interface I is located at the N-SOSSA_N_ tail, forming a C-shaped cavity, wherein Thr40 and Leu42 interact with Leu61 and Ile62 of hSSB1, respectively. Leu42 and Ala44 of INTS3 also form a backbone H-bond interaction with Lys94 of hSSB1. At interface II (C-SOSSA_N_), hydrophobic interactions exist between grooves formed by Phe315, Tyr328, and Trp331 of INTS3 and patches formed by Ile22, Pro64, Phe98, Val101, and Tyr102 of hSSB1. Other prominent interactions at this interface include a salt bridge, ionic bond, and H-bond. The opposite side of interface I of INTS3 forms a shallow concave groove meant for SOSS-C binding. The INTS3 interacts with SOSS-C mainly via H-bonds buried with an accessible surface area of 2470 Å^2^.^17^

A previous study has demonstrated that hSSB1 binds to p21 and prevents its ubiquitin-mediated proteasomal degradation. In human hepatocellular carcinoma (HCC), overexpressed hSSB1 positively regulates the activity of p21 to modulate its cell cycle activity.^18^ The involvement of upregulated activity of the p21 function has been suggested to contribute to the development of resistance of cancer cells to chemotherapy and radiotherapy.^19^ hSSB1 also interacts with p53 and protects it from proteolytic degradation.^20^ hSSB1 is overexpressed in different types of cancers including nonsmall cell lung cancer (NSCLC), HCC, and prostate cancer,^21^ and its involvement in the modulation of the activities of proteins participating in DNA damage repair, cell cycle progression, and checkpoint regulation makes it a promising therapeutic target for cancer.^18,22^ The upregulated hSSB1-mediated DNA repair activity also makes the cancer cells resistant to traditional therapeutic modalities, and the studies have shown that knocking down of hSSB1 makes the resistant tumor cells sensitive to conventional therapies such as chemo- and radiotherapy.^23,24^

While previous studies have investigated the inhibition of ssDNA binding activity of hSSB1 by targeting its OB-fold using oligonucleotides^25,26^ and small molecules,^27^ the prospect of inhibiting hSSB1 by disrupting its interaction with its crucial binding partner in the heterotrimeric SOSS1 complex, INTS3, remains unexplored. While targeting the OB-fold lacks specificity toward hSSB1, interrupting the PPI interface between hSSB1 and INTS3 might have therapeutic potential to exploit the DNA damage response, particularly in oncology settings. As such, we used a structure-based virtual screening protocol to perform molecular docking of a specific PPI library from Enamine followed by *in vitro* validation of a small set of top hits for their PPI disruption capabilities in U2OS cells and effects thereafter, on DNA damage repair. Our study suggests potential PPI disruption by three of the virtually screened hits, preventing the recruitment of these proteins to DNA-damaged sites.

## Results

### Identification of Compounds that Interfere with Association between INTS3 and hSSB1

**Figure 2:**
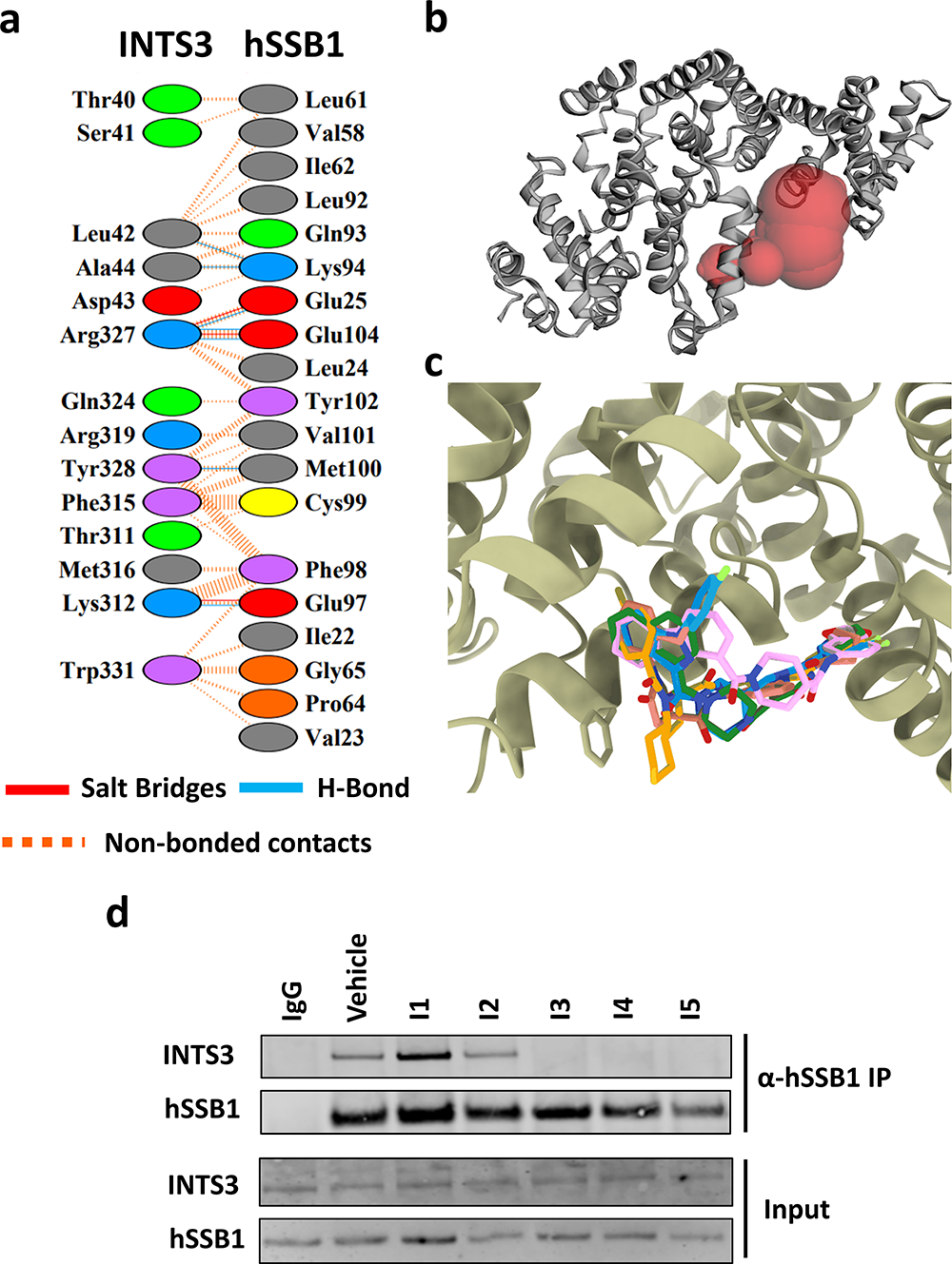
Targeting the INTS3-hSSB1 interface with small molecule inhibitors.(a) PPI residues between INTS3 and hSSB1 from the crystal structure, PDB ID: 4OWW, generated by PDBsum indicate the involvement of interaction via formation of the salt bridge, H-bonds, and nonbonded interaction. (b) Ligand binding cavity on the N-terminus of INTS3 predicted by the CASTp 3.0 server. (c) Overlaid binding poses of the five top-scoring ligands (green: **I4** - Z826681950, pink: **I3** - Z336696716, orange: **I5** - Z978928456, blue: **I1** - Z1033202002, and salmon: **I2** - Z30413849) with INTS3 from virtual screening using Autodock Vina. (d) Co-immunoprecipitation assay (*n* = 3) of endogenous INTS3 with hSSB1 from U2OS cell lysates after compound (**I1**–**I5**) treatment for 36 h.

To identify the interacting amino acid residues between INTS3 and hSSB1, the three-dimensional (3D) X-ray crystal structure of the SOSS1 complex (PDB ID: 4OWW) was investigated. The amino acid residues of hSSB1 interact with INTS3 via the H-bond, salt bridge formation, and hydrophobic interactions, represented in Figure 2a. Investigation of the INTS3 surface indicates the availability of a surface cavity at the hSSB1 binding interface with an area of 151.08 Å^2^ (Figure 2b). Enamine’s PPI compound library was virtually screened against the 3D structure of INTS3 at the hSSB1 binding interface using Autodock Vina via Parallelized Open Babel and Autodock suite Pipeline (POAP).^28^ The top 400 compounds binding to INTS3 at its N-terminus were obtained from the virtual screening and were subjected to further processing. The ranking of the compounds was compared with the docking protocols having exhaustiveness values of 8 and 16. With exhaustiveness values of 8 and 16, the compounds were ranked similarly. Since the sampling and scoring used in the Autodock Vina docking program were not validated for the INTS3 compounds, only the top five compounds (Z826681950, Z336696716, Z978928456, Z1033202002, and Z30413849) mentioned in Table 1 with a docking score of −10 kcal/mol from the virtual screening of the Enamine PPI library were further investigated using *in vitro* methods. Investigation of the docked pose of the protein–ligand complex (Figure 2c) shows the binding of the top-scoring ligands at the groove between the α-helical region of the N-terminus of INTS3 (N-SOSSA_N_).

**Table 1:**
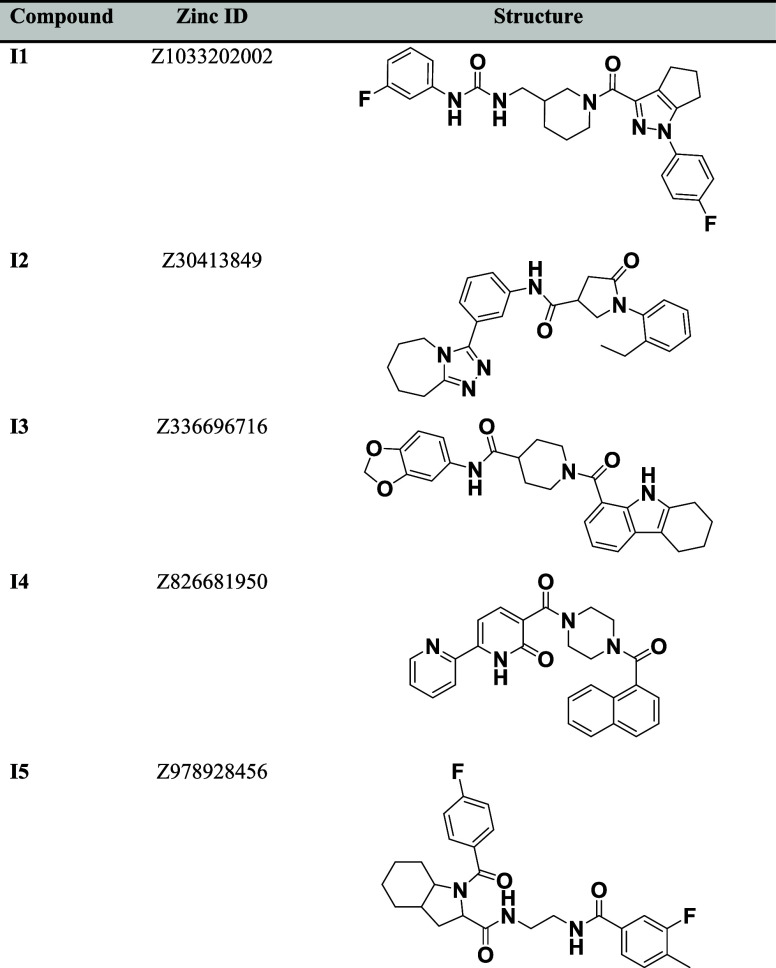
Two-Dimensional (2D) Structures of the Top Five Compounds from the Virtual Screening of the Enamine PPI Library against INTS3

To determine whether the compounds identified by *in silico* approaches had an impact on the association between INTS3 and hSSB1, we performed hSSB1 immunoprecipitation analysis on lysates collected from U2OS cells treated with or without the five compounds (termed **I1**–**I5**) for 36 h. As shown in Figure 2d, compounds **I3**, **I4**, and **I5** resulted in a reduced association between INTS3 with hSSB1, compared with the vehicle. Compounds **I1** and **I2** did not reduce the association between INTS3 and hSSB1. These findings point to the possibility that at least three compounds might interrupt the association between INTS3 and hSSB1.

### Proof-of-Concept of Cell-Based Approaches to Assess the Reduced Association between INTS3 and hSSB1

**Figure 3:**
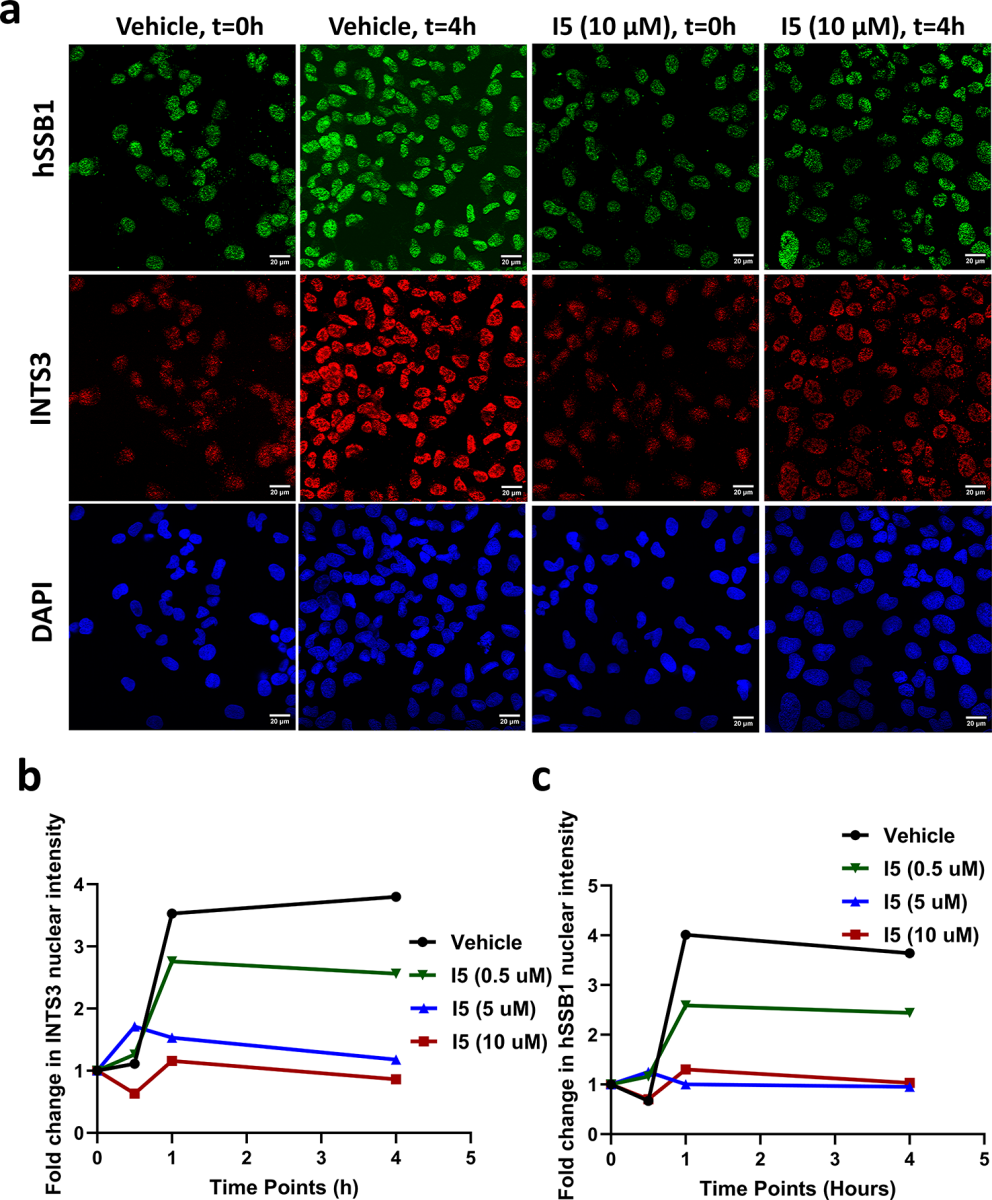
Effect of compound **I5** treatment on the recruitment of INTS3 and hSSB1 to DNA damage sites. The U2OS cells were irradiated with 6 Gy of IR post-treatment with compound **I5** and immunostained at different time points (0, 0.5, 1, and 4 h). (a) Representative figure (20 μm) of cells stained with DAPI (blue), indicating that the nucleus shows a decrease in fluorescence intensity of **I5** treated cells after 4 h at 10 μM compared to DMSO treatment as a vehicle. (b, c) Plot of fold change in INTS3 and hSSB1 nuclear intensity shows a decrease after an hour in **I5** (5 and 10 μM) treated cells, unlike vehicle-treated cells.

Having identified compounds that reduce the association between INTS3 and hSSB1, we next sought to determine whether these compounds impact the recruitment of these two proteins to DNA damage sites by quantifying the number of nuclear INTS3 and hSSB1 foci in cells post-IR by using immunofluorescence. Given INTS3 and hSSB1, relocating to the site of DNA damage following IR-induced DNA damage,^11^ INTS3 and hSSB1 immunofluorescence were performed on cells treated with or without the compounds **I3**, **I4**, and **I5** following IR. Immunofluorescence was performed on pre-extracted cells to remove soluble proteins and enable imaging of proteins bound to insoluble structures such as chromatin. As shown in Figure 3a–c, IR induced the recruitment of INTS3 and hSSB1 (vehicle) to chromatin, consistent with earlier findings. At the 10 μM concentration of **I5**, the chromatin localization of INTS3 and hSSB1 was reduced after 30 min. However, 0.5 and 5 μM concentrations of compound **I5** markedly reduced the chromatin localization of both INTS3 and hSSB1 within 1 h of treatment and at 4 h following IR. Compounds **I3** and **I4** did not display any reduction in the INTS3 and hSSB1 focus formation compared to the vehicle-treated cells. These data suggest that compound **I5** impacts the efficient recruitment of INTS3 and hSSB1 to the chromatin following IR-induced DNA damage.

### Molecular Dynamics Simulations of the Five Tested Compounds with INTS3

**Figure 4:**
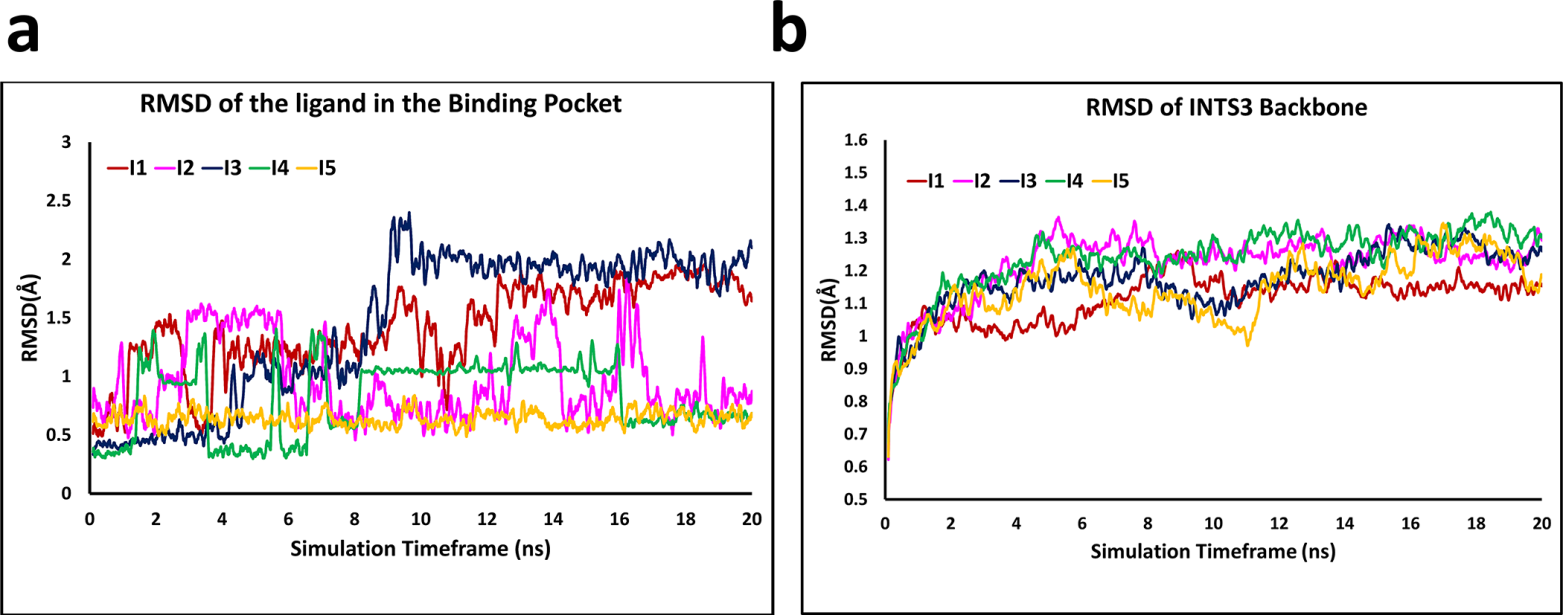
Plot showing moving average of root mean square deviation (RMSD) vs time of the INTS3-ligand complex. (a) Average RMSD plot of the ligands (**I1**–**I5**) bound to the receptor and (b) RMSD-time plot (moving average) of INTS3 backbone residues over a simulation time frame of 20 ns, obtained from the single simulation trajectories.

The molecular dynamics (MD) simulations were carried out to understand the conformational transition, dynamic behavior, and stability of the ligand at the protein’s binding pocket. The stability of each MD simulation trajectory was assessed by monitoring the root mean squared deviation (RMSD) of the ligands (**I1**–**I5**) and receptor, as shown in Figure 4. In Figure 4a, ligand **I5** can be seen to acquire a stable conformation throughout the simulation time frame when compared to **I4**. The ligand **I2** was also found to be stable and achieved an RSMD value of ≤1.5 Å. In the case of two ligands, **I1** and **I3**, the RMSD slightly increased to ∼2 Å after 10 ns of the classical MD simulation. Overall, all of the compounds were stable in the receptor-binding pocket. The receptor’s backbone atoms were relatively stable as compared to the ligand molecules with an RMSD of ≤1.3 Å (Figure 4b). This explains the stability of the receptor pocket upon binding to the ligands. Further evaluation of root mean square fluctuations (RMSF) of INTS3 atoms in its apo-state and when bound to each ligand was evaluated to understand the conformational flexibility of the receptor pocket upon binding to the ligands. From Figure S1 (Supporting Information), it can be seen that the binding of compounds **I1**–**I5** increased the overall stability of INTS3, as indicated by the decrease in the RMSF values. However, INTS3 bound to compounds **I1** and **I2** exhibited conformational changes across groove 2 (residues 130–136) and groove 1 (residues 320–335) regions, respectively, when compared to the **I5** bound to INTS3. Also, the receptor bound to compound **I3** displayed relatively higher conformational flexibility in the groove 2 region and the region between grooves 1 and 2.

**Figure 5:**
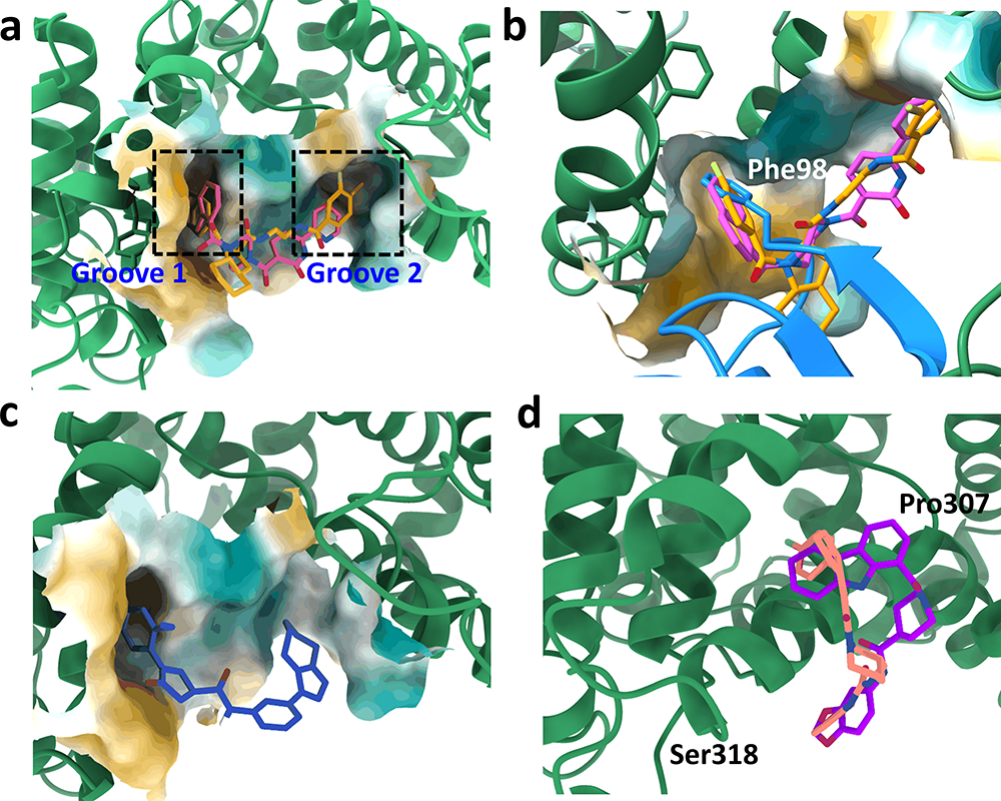
Binding mode of screened compounds to the INTS3 pocket. The representative frame from the top cluster from the MD simulations of (a) compounds **I4** (pink) and **I5** (yellow) overlaid at the binding site of INTS3 (green ribbon) shows interaction with amino acid residues at two binding grooves, and (b) the terminal *N*-naphthaloyl ring of **I4** and *p*-fluoro benzoyl ring of **I5** share the same binding pocket as the aromatic ring of Phe98 of hSSB1 (blue); (c) compound **I2** also shares similar binding pockets as **I4** and **I5**; however, pocket 2 is shallow. (d) Compounds **I1** (salmon) and **I3** (purple) wrap around α-helix 1 from the residue (Pro307–Ser318).

The representative frame from the most abundant cluster of 20 ns production simulation trajectories of each system (Figure 5) shows occupancy of two grooves of INTS3, grooves 1 and 2, by compounds **I4** and **I5**. These grooves are located between the α-helices of the N-terminus of INTS3 at the hSSB1 binding interface II (Figure 5a). Investigation of other clusters indicates the formation of a stable INTS3-**I4**/**I5** complex at these two binding sites. Interestingly, when these binding poses of two ligands were overlaid with the hSSB1 binding from the crystal structure (PDB ID: 4OWW), the terminal *N*-naphthaloyl ring of **I4** and *p*-fluoro benzoyl ring of **I5** were found to occupy the INTS3 binding site at groove 1 that is occupied by Phe98 of hSSB1 (represented in Figure 5b). Although the representative frame from the top cluster of **I2** also occupies a similar binding site as compounds **I4** and **I5** (Figure 5c), investigation of the simulation trajectories showed high flexibility of the ligand at the groove 2 region. Compounds, **I1** and **I3**, unlike other compounds, instead of occupying the second binding groove, were shown to wrap around the α-helical structure (Pro307–Ser318) at the hSSB1 binding interface II (Figure 5d).

**Figure 6:**
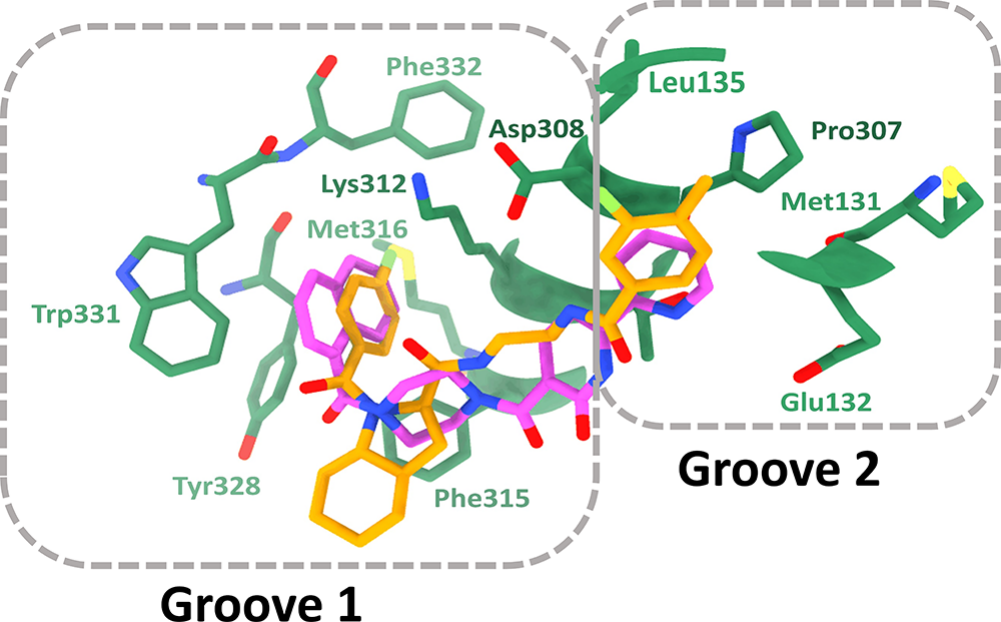
Interactions of compounds **I4** and **I5** from the Enamine PPI library. 3D model of interacting residues of INTS3 (green) with the overlaid structures of compounds **I4** (pink) and **I5** (yellow).

From the top five compounds identified through the virtual screening, the interactions of **I4** and **I5** at the binding pocket of INTS3 showed engagement with the N-terminal helical residues, Asp308, Lys312, Phe315, Meth316, Tyr328, Trp331, and Phe332 at groove 1 (Figure 6). These important amino acid residues of hSSB1 form a PPI interface with INTS3 via H-bond and hydrophobic contacts (Figure 2a). At groove 2, these two compounds containing the aromatic ring interact with Met131, Glu132, Leu135, and Pro307 via H-bond and hydrophobic interactions. The overlaid structures of these two compounds show the occupancy of two binding pockets of INTS3 via aromatic heterocyclic rings, which are attached by bulkier linker groups (Figure 6).

### MM-GBSA Calculations of INTS3-Ligand Binding Free Energies

The rescoring of docked complexes was considered to investigate the correlation between *in silico* screening and the *in vitro* experimental results. For this, the docked pose of each ligand (**I1**–**I5**) was rescored using MM-GBSA calculation (eq 2) after simulating them for 20 ns at NPT ensembles. Previous benchmarking studies have shown the better performance of short MD simulation of protein–protein and protein–ligand complexes over the enhanced sampling for MM-GBSA-based free energy calculation.^29,30^ From the relative binding free energies (Δ*G*_Total_) of the systems (indicated in Table 2), it can be inferred that ligands **I4** and **I5** possess the strongest binding with the INTS3 receptor pockets, with energies of −8.91 and −7.75 kcal/mol, respectively, as compared to other compounds. Although the difference in the energy contribution from the hydrophobic interactions (*E*_VdW_) (−1.93 kcal/mol) and the contribution from the combined electrostatic (*E*_Elec_) and polar solvation (*E*_GB_) energies (0.38 kcal/mol) between these two systems are negligible, the overall difference in Δ*G*_Total_ is due to the change in entropy (*T*Δ*S*) of the system. Among compounds **I1**, **I2**, and **I3**, compounds **I1** and **I2** possess relatively higher binding free energies owing to their better hydrophobicity compared to **I3**. However, when *T*Δ*S* values of each ligand bound to the systems are compared, compound **I2** displayed maximum randomness indicated by the least negative value of −20.90 kcal/mol within considered simulation time frames.

**Table 2:** Rescoring of Ligands Using MM-GBSA-Based Free Energy Calculation^a^

		**free energy of binding** (kcal/mol)								
**Sr. no**	**ID**	**E**_**VdW**_	**E**_**Elec**_	**E**_**GB**_	**E**_**Surf**_	**G**_**Gas**_	**G**_**Solv**_	**Δ***G*_**Bind**_**(mean ± SD)**	**TΔ***S***(mean ± SD)**	**Δ***G*_**Total**_**(mean ± SD)**
1	**I1**	–40.56	–9.66	26.36	–5.02	–50.21	21.34	–28.87 ± 1.12	–22.60 ± 1.67	–6.27 ±1.45
2	**I2**	–38.04	–5.24	21.28	–4.55	–43.28	16.73	–26.54 ± 0.18	–20.90 ± 0.61	–5.64 ± 0.53
3	**I3**	–37.06	–7.17	24.60	–4.32	–44.23	20.28	–23.95 ± 1.71	–21.18 ± 0.86	–2.76 ± 1.00
4	**I4**	–43.80	–22.55	40.82	–5.30	–66.35	35.53	–30.82 ± 2.73	–21.92 ± 1.73	–8.91 ± 1.32
5	**I5**	–45.73	–16.28	34.93	–5.67	–62.01	29.26	–32.75 ± 0.26	–24.90 ± 0.16	–7.85 ± 0.19

a Values represent the average of each energy term from a triplicate simulation of 20 ns each. SD (±) represents the standard deviation of the Δ*G*_Bind_, *T*Δ*S*, and Δ*G*_Total_ from three simulations.

**Figure 7:**
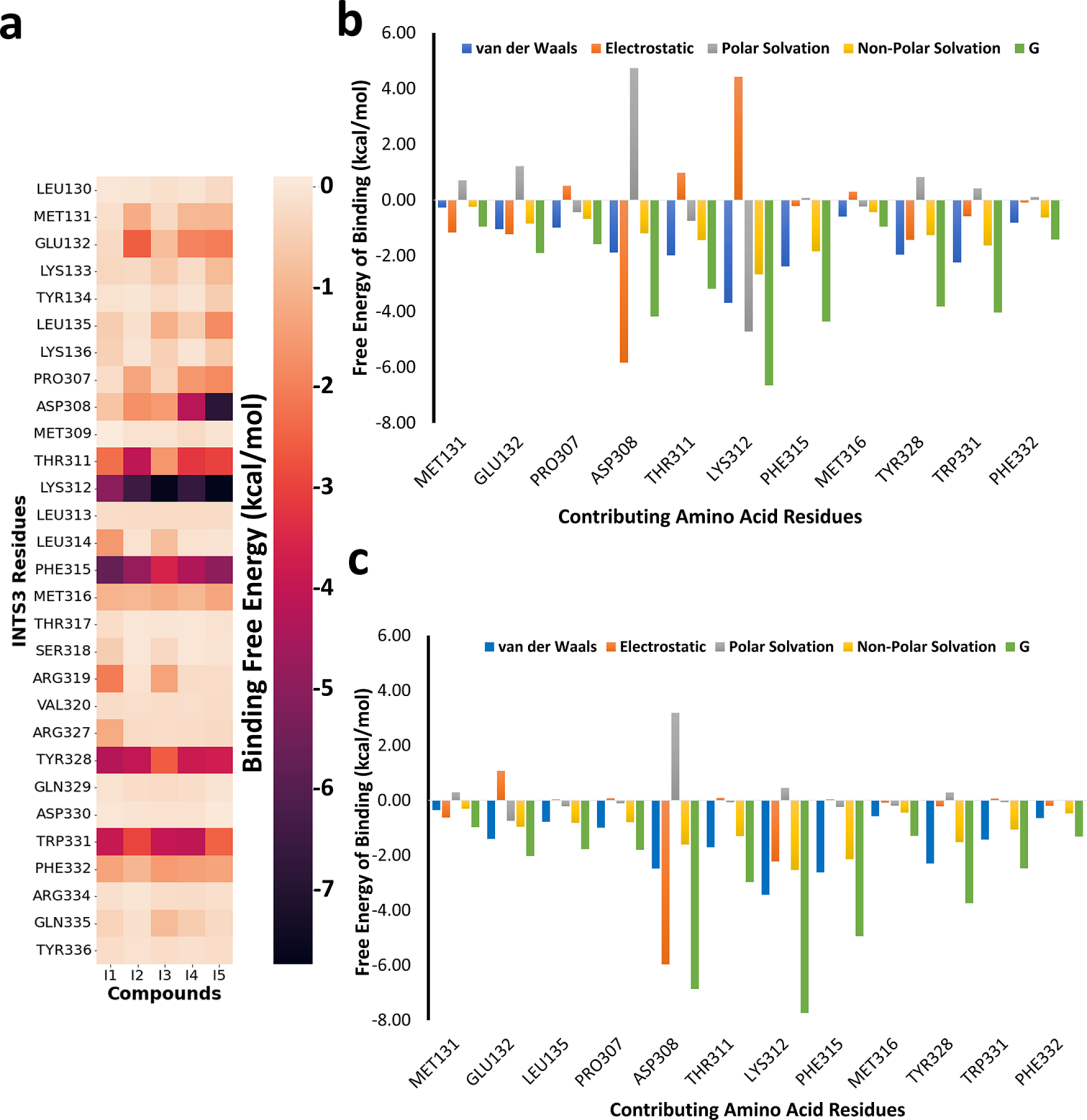
MM-GBSA free energy decomposition of compounds binding to INTS3. (a) Heatmap of pairwise decomposition of relative binding free energies of interacting amino acid residues within 6 Å of ligands; decomposition of the binding free energy on a per-residue basis into contributions from electrostatic interactions, vdW energy, and polar and non-polar solvation energies in the (b) INTS3-**I4** complex and (c) INTS3-**15** complex.

Insights into pairwise MM-GBSA free energy decompositions (Figure 7a) of the INTS3 residues within 6 Å of the five compounds explain the role of contributing amino acids to the total energy. From the heatmap, it can be seen that Asp308, Thr311, Lys312, Phe315, Met316, Tyr328, and Trp331 of the N-terminus of INTS3 are major residues mediating interactions with the ligands. These residues are crucial INTS3 residues responsible for PPI with hSSB1. Among these, Lys312 mediates the strongest interaction with all the compounds with binding free energies of −5.04 to −7.74 kcal/mol followed by Phe315, which has a binding free energy from −3.59 to −5.69 kcal/mol and other residues like Try328 and Trp331. Asp308 prominently interacts with **I4** (−4.18 kcal/mol) and **I5** (−4.18 kcal/mol) and displays weaker interaction with the other three compounds. Compounds **I2**, **I4**, and **I5** also interact with the residues in the region Met131-Lys136, with Met131 and Glu132 being the major contributors of interaction. However, in the case of **I1** and **I3**, these interactions are missing. Instead, the ligands engage themselves with other residues like Leu314, Arg319, and Arg327 (**I1** only) with weaker binding free energies of less than 2 kcal/mol.

To obtain detailed information about the hot-spot residues of **I4** and **I5**, the total binding free energy (*G*) of some key residues is decomposed into their contributing energy components, such as vdW interactions, the electrostatic energies, and the polar and the nonpolar solvation energies (Figure 7b,c). In compound **I4** (Figure 7b), vdW interactions and nonpolar solvation energies of all the interacting residues displayed favorable contributions toward the *G* values. Generally, the electrostatic and polar solvation energies compensate for each other. All the amino acid residues, except Pro307, Thr311, Met316, and Phe332, displayed a favorable contribution from the sum of electrostatic and polar solvation components. Although Lys312 also displayed a positive electrostatic energy of 4.42 kcal/mol like Thr311, this was compensated by a negative polar solvation energy term, −4.72 kcal/mol. Again, the nonpolar solvation and vdW terms majorly contributed to the Δ*G* values of all the hot-spot residues of compound **I5** (Figure 7c). Among these residues, the Asp308 and Lys312 displayed increased contribution from the favorable electrostatic interaction with energies, −5.97 and −2.22 kcal/mol, respectively, over the polar solvation energies, 3.19 and 0.46 kcal/mol. For residues, such as Glu132, Pro307, Thr311, Phe315, Tyr328, and Trp331, the unfavorable electrostatic interactions were compensated by the polar solvation terms.

## Discussion

Previous studies have reported the overexpression of hSSB1 in different cancers, including HCC, NSCLC, and prostate cancer, and its role in checkpoint activation, radiosensitivity, and genomic instability.^11,18,20^ Depletion of hSSB1 via the SCF Ubiquitin E3 complex, F-box containing protein, Fbxl5, or hSSB1 knockdown and/or depletion of its binding partner in the SOSS1 complex, INTS3, yields impaired or defective DNA repair and increased sensitivity of cells to IR,^11,14−16,19,31^ suggesting that targeting the INTS3 and hSSB1 with inhibitors to destabilize the interaction between the two proteins might resensitize tumor cells to the chemo- and/or radiotherapeutics. Recent developments in medicinal chemistry, biochemistry, molecular biology, and biophysics have enabled the further exploration of PPIs in the modulation of biological functions. Several PPIs involved in different types of DNA damage response pathways have been explored for the identification of chemotherapeutic agents inducing synthetic lethality in cells, sensitizing the cells to DNA damaging agents, and modulating cell cycle or apoptotic proteins.^32^ Although the INTS3-hSSB1 binding interface has never been studied, the availability of the 3D X-ray crystal structure as the SOSS1 complex opened the opportunity to adapt the structure-based drug design scheme.^33^

We explored a structure-based design to screen for inhibitors disrupting the INTS3-hSSB1 binding interface. The INTS3 binding surface was considered due to the availability of a well-defined cavity at its PPI interface. Although the high-throughput virtual screening of large libraries provides technological freedom to explore the vastness and diversity of the chemical space, the major challenges associated with such a big library are a higher probability of errors while ranking them, inaccuracy in the prediction of docked poses, and missing some protein–ligand interactions from a small fraction of the ultralarge data set.^34^ Therefore, the selection of a small but specific PPI library from Enamine, for the high-throughput virtual screening (HTVS) against the INTS3 binding pocket, enabled the identification of 400 hit molecules binding to the “hot-spot” residues. Since the Autodock Vina has not been benchmarked for the protein target considered in the study, the identified hits via *in silico* screening were considered for validation using the *in vitro* assays.

Due to the high risk of failure associated with the screening of compounds against the novel PPI target, a pilot study was conducted using the top five virtually screened hits for initial validation using *in vitro* assays. For this, the co-immunoprecipitation technique was used to evaluate the *in vitro* disruption of PPI between INTS3-hSSB1 in the U2OS cell lines. U2OS cells are widely studied for measurements of genomic instability and are well-known for expressing DNA damage repair proteins.^35,36^ Three of the five tested compounds were found to reduce the association between INTS3-hSSB1 and U2OS cells, indicating the success of the *in silico* screening protocol. However, there are some limitations to our observations. For example, more validations need to be carried out using biophysical techniques such as surface plasmon resonance or isothermal titration calorimetry to demonstrate the direct binding of compounds with the INTS3 binding pocket. Also, the *in vitro* disruption of PPI between INTS3 and hSSB1 needs to be investigated further using recombinant proteins to examine whether they disrupt the direct interaction of these proteins rather than through another indirect mechanism. Further cell-based immunofluorescence staining indicates that the interference of association of two binding partners by **I5** prevented their accumulation at DNA damage sites following IR-induced DNA damage. Although **I3** and **I4** were also capable of disrupting the association between INTS3 and hSSB1, only **I5** displayed impacts on DNA repair, suggesting a requirement for further investigation to understand their role at the functional level. Overall, these findings will have interest in the DNA damage repair field to better understand INTS3 and hSSB1 biology.

The detailed investigation of the binding mode of the active compounds using the MD simulation studies and pairwise energy decomposition suggest their possible INTS3-hSSB1 PPI disruption by preventing the interaction of Phe98 and other residues such as Cys99 and Glu97 of the hSSB1 with the hot-spot residues of INTS3 at binding interface II and groove 1. This is in alignment with a previous mutagenesis study reporting the essential role of Phe98 of hSSB1 at interface II for INTS3 binding.^17^ Although groove 2 of INTS3 is not directly involved in the interaction with hSSB1, indulgence of compounds in this cavity imparts conformational stability to the bound ligands. Collectively, these data validate the *in silico* findings and provide further scope for structural optimization of the hit compound, **I5**, for the identification of lead. Calculating the hit rates for the drug discovery methods, such as experimental high-throughput screening (HTS) and virtual screening, provides a rough estimate of the success of the method. Studies have shown that the implementation of virtual screening protocols yields a better hit rate than traditional HTS.^37,38^ Although, as an investigatory approach, we could identify some novel molecular scaffolds that might disrupt the INTS3-hSSB1 interactions; however, this data set is too small to assess the hit rate and quantify the effectiveness of the Autodock Vina-based virtual screening protocol for INTS3 binding. Therefore, this study warrants further assessment of the Enamine PPI library as a potential inhibitor of INTS3-hSSB1 using the *in vitro* biophysical, biochemical, and cell-based assays specific to INTS3.

## Conclusions

In this section, a novel approach has been adopted to modulate the function of the SOSS1 complex via targeting INTS3. Considering the structural and functional importance of the key adapter of the complex, INTS3, the PPI interface at INTS3 and hSSB1, distinct from the oligonucleotide binding surface of the OB-Fold of hSSB1, was considered for the identification of potential inhibitors. Using virtual screening combined with *in vitro* cell-based assays and classical MD simulation, three hit molecules have been identified to interfere with the PPI between the INTS3-hSSB1 complex. Disruption of PPI between this complex and one of the tested compounds prevented the recruitment of the proteins to the DNA damage site. This pilot study identified three novel small molecule scaffolds to inhibit INTS3-hSSB1 PPI via targeting INTS3 in its N-terminal PPI cavity. This opens the scope to explore these molecules for further structural optimization and targeting this complex for the discovery of novel chemotherapeutic agents.

## Methods

### Identification of the Ligand Binding Pocket at the PPI Interface of INTS3-hSSB1

To visualize the amino acids involved in PPI between INTS3 and hSSB1, the PDB structure of the SOSS1 complex (PDB ID: 4OWW) was subjected to analysis using the PDBsum web server (http://www.ebi.ac.uk/thornton-srv/databases/pdbsum/) from EMBL-EBI.^39^ The surface pocket of INTS3 was predicted using the CASTp 3.0 web server (http://sts.bioe.uic.edu/castp/).^40^

### *In Silico* Screening of the PPI Library

#### Protein and Ligand Preparation

The refined 3D X-ray crystallographic structure of the human SOSS1 heterotrimeric complex with the 35nt ssDNA (PDB ID: 4OWW) having a resolution of 2.30 Å was retrieved from the PDB-REDO web server (https://pdb-redo.eu/). The protein preparation steps were carried out using UCSF Chimera v1.14.^41^ The chains containing hSSB1 (chain B), C9ORF80 (chain C), and ssDNA (chain D) were deleted. The water molecules were deleted, and polar hydrogens and Gasteiger partial charges were added using the Dock Prep module. The structure of the INTS3 chain was checked for any missing residues and mutations using Uniport ID: Q9NRY2 as the query sequence. Considering the PPI residues between hSSB1 and the N-terminus of INTS3 (at interfaces I and II), a centroid was defined with a 2.0 Å radius of the interacting amino acid residues. A grid box was generated with dimensions of 35 Å × 35 Å × 40 Å and *XYZ* coordinates of 37 Å × 101 Å × 26 from the center, covering interfaces I and II.

The large-scale virtual screening has mostly been used in drug discovery due to the higher probability of finding hits from a wider variety of chemical scaffolds.^42^ However, this has a higher probability of obtaining false negatives. On the other hand, virtual screening has successfully identified glucocorticoid receptor antagonists and fibrinolysis inhibitors from a modest library.^43^ Motivated by this, a specific PPI library from Enamine (https://enamine.net/compound-libraries/targeted-libraries/ppi-library), consisting of 40,640 diverse sets of compounds with protein domain affinity and lead-like properties, passing PAINS filters were considered for the virtual screening for INTS3 binding. These compounds were subjected to the ligand preparation module (POAP_lig.bash script) of Parallelized Open Babel and Autodock suite Pipeline (POAP).^28^ POAP offers several advantages, e.g., geometry optimization and 3D conformer generation, and filters the erroneous data set for flawless operation. In the ligand preparation step, OpenBabel was utilized to convert 2D input files from SDF format to 3D coordinates. Conformers were generated by using the Weighted Rotor search method to produce 100 conformations for each ligand. Energy minimization was performed using 5000 steps of the conjugate gradient method using the MMFF94 force field. Convergence criteria, van der Waals (vdW), and electrostatic cutoff distances were set to default values of 1 × 10^–6^, 6.0, and 10.0 Å, respectively. Hydrogens were added, 11 erroneous files were removed, and finally, 40,629 output files of ligands were generated in pdbqt format.

#### Virtual Screening of the Enamine PPI Library

The virtual screening of 40,629 compounds from the Enamine PPI library against the INTS3 protein structure was carried out using the virtual screening module (POAP_vsbash script) of POAP,^28^ enabling the automation of the Autodock Vina-driven screening process. For docking of the ligands, the grid box of dimensions mentioned in the above section was used, and the exhaustiveness was set to 8 and 16. The ligands were ranked based on the binding energies (in kcal/mol), with the most negative at the top, indicating a stronger protein–ligand interaction. A total of three repeats of the virtual screening were considered. To validate the *in silico* findings, the five highest-scoring compounds from the virtual screening of the PPI library against INTS3 using Autodock Vina were purchased from Enamine, Ukraine (https://enamine.net).

#### Molecular Dynamics Simulation of the Top Five Hits from Virtual Screening

The top-scoring docked poses of the top five ligands from the Autodock Vina screening were subjected to classical MD simulation in the presence of protein, explicit solvent, and ions. The initial input files for ligands were prepared using the Antechamber module^44^ of AmberTools18.^45^ Partial charges for the ligands were derived using the AM1-BCC charge model.^46^ The molecular system for the simulation was prepared using the tleap program, where Amber ff14SB,^47^ GAFF2,^48^ and TIP3P^49^ were used as protein, ligands, and water force fields, respectively, with a default PBRadii set to the “mbondi3”. The protein–ligand complexes were solvated using the TIP3BOX water model within a 12.0 Å octahedral box from the protein surface. The system was neutralized by adding either Na^+^ or Cl^–^ ions followed by further adding equimolar quantities of Na^+^ and Cl^–^ ions to get the final salt concentration of 0.15 M.

All the MD simulations were carried out using the PMEMD.CUDA module of Amber16.^50,51^ The nonbonded interaction cutoff was set to 12 Å during energy minimization and simulation steps. The initial minimization of the system was performed for 5000 steps at 298.15 K using 2500 steps of the steepest descent and the remaining 2500 steps of the conjugate gradient method. The optimized structure was then heated from 100.15 to 298.15 K for 0.5 ns (ns) at the constant number, constant volume, and temperature (NVT) ensemble with restraints of 5.0 kcal/(mol Å^2^) on the protein–ligand complex and equilibrated for 1 ns under this condition. Another 1 ns equilibration was performed under constant number, pressure, and temperature (NPT) conditions by restraining the protein–ligand complex with 5.0 kcal/(mol Å^2^). Gradually, the positional restraints were reduced from the protein–ligand complex and the final 1 ns equilibration was performed by putting weak positional restraints of 0.1 kcal/(mol Å^2^) on protein backbone atoms. The long-range electrostatic interactions were evaluated using the particle mesh Ewald (PME) method.^52,53^ The complex was subjected to a final 25 ns production simulation at the NPT ensemble maintained at 298.15 K, using a Langevin thermostat with a collision frequency of 2 ps^–1^. Pressure was regulated using isotropic pressure scaling with a relaxation time of 0.5 ps. A weak Berendsen coupling method was used to maintain the temperature and pressure of the system.^54^ The SHAKE algorithm^55^ was applied over an integration time of 2 fs (fs). The trajectories of the initial 5 ns were considered as equilibration steps, and hence, the final 20 ns MD production steps consisting of 2000 frames captured at an interval of 10 ps (ps) were used for final analysis using the CPPTRAJ module^56^ of AmberTools 18.^45^

#### Relative Free Energy of Binding Using the Molecular Mechanics-Generalized Born Surface Area (MM-GBSA) Method

In molecular docking, scoring functions were employed to evaluate the docking poses generated by conformational searches. These scoring functions consider various approximations to simplify the calculations to achieve the speed in high-throughput screening, thereby compromising the accuracy of the calculations. This makes molecular docking predict incorrect binding energies when compared to the experimental values.^57^ Therefore, rescoring the docking poses provides a better estimation of binding energetics, Δ*G*_bind_, determined in eqs 1 and 2*.* Studies have shown that MM-GBSA outperforms MM-PBSA in terms of computational accuracy and has been widely used for free energy calculation due to the relatively faster computational speed than the PBSA method.^58,59^
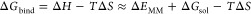
1
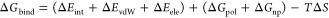
2where Δ*H* in eq 1 represents the changes in enthalpy and *T* and *S* are the absolute temperature and an entropy estimate, respectively. Δ*E*_MM_ is the change in gas phase molecular mechanics (MM) energy, which includes internal energy (Δ*E*_int_) (bond, angle, and dihedral energies), electrostatic energy (Δ*E*_ele_), and van der Waals energies (Δ*E*_vdw_). Δ*G*_sol_ is the sum of the contribution of electrostatic solvation energy (polar contribution), Δ*G*_pol_, and nonelectrostatic solvation component (nonpolar contribution), Δ*G*_np_. The polar contribution is calculated using either the GB or PB model. The MM-GBSA relative free energy of binding on MD trajectory containing the last 2000 frames was calculated for each protein–ligand complex using the MMPBSA.py module^60^ of AmberTools18.^45^ The GB model, GBneck2^61^ (igb = 8), was used for the calculation of the polar solvation energy component, and the salt concentration was set to 150 mM. The nonpolar solvation energy was estimated using the solvent-accessible surface area (SASA)^62^ using the fast LCPO algorithm.^63^ The dielectric constant for the solvent was set to 80, and a solute dielectric constant of 1 was used. The entropy of each system containing 150 frames was calculated using normal-mode analysis (NMA) with a default convergence cutoff value of 0.001 kcal mol^–1^ Å^–1^^64^ at 298.15 K.

### *In Vitro* Validation of the Top Hits

#### Antibodies and Reagents

The primary antibody against INTS3 (antirabbit, cat no. A302-050A, Bethyl) was purchased from Thermo Fisher Scientific, and an antigoat IgG isotype control antibody was purchased from Sigma-Aldrich. The antigoat hSSB1 antibody was generated in-house as described previously.^65^ The secondary antibodies, donkey antirabbit (cat no. 926-68073) and donkey antigoat (cat no. 926-62214), were from LI-COR, Inc. The secondary antibodies for immunofluorescence, Alexa Fluor 488 donkey antisheep (Invitrogen, cat no. A11015), Alexa Fluor 488 donkey antimouse (Invitrogen, cat no. A21202), and Alexa Fluor 594 donkey antirabbit (Invitrogen, cat no. A23753) antibodies, were purchased from Life Technologies. 4′-6-Diamidino-2-phenylindole (DAPI) was from Life Technologies. A complete EDTA-free protease inhibitor mixture was obtained from Roche Applied Sciences. The Pierce bicinchoninic acid (BCA) protein assay kit (cat no. 23225) was purchased from Thermo Fisher Scientific.

#### Cell Culture and Cell Treatments

The U2OS cell line was obtained from the American Type Culture Collection (ATCC) and maintained in the Glibco RPMI-1640-medium + l-glutamine (cat no. 11875-093, Life Technologies), supplemented with 10% fetal bovine serum (FBS, Sigma-Aldrich). Cells were cultured at 37 °C in a humidified 5% CO_2_ atm. For *in vitro* compound treatments prior to immunoprecipitation and western blot analyses, cells seeded on a Petri dish were treated with compounds **I1**–**I5** at a concentration of 10 μM. After 24 h of treatment, the culture media were replaced with the fresh media containing the compounds with the mentioned concentration and left for another 12 h.

#### Collection of Lysates, Immunoprecipitation, and Western Blot Analyses

For the whole cell lysate collection, the U2OS cells were washed with phosphate-buffered saline (PBS) and lysed in lysis buffer (50 mM HEPES-pH 7.5, 150 mM KCl, 5 mM EDTA, 0.05% IGEPAL CA-630 (v/v), 1× protease inhibitor cocktail (Roche), and 1× phosphatase inhibitor cocktail (Cell Signaling Technology)). The lysates were sonicated and centrifuged to remove extracellular debris. Using the supernatants, the total protein yields were determined using the BCA protein assay. The total protein (20 μg) samples were denatured in 2× Laemmli buffer supplemented with 8% β-mercaptoethanol for 8 min at 80 °C.

For immunoprecipitation, protein samples were prepared with 400 μg of protein in 400 μL of lysis buffer. Lysates were incubated overnight with 3 μg of the hSSB1 antibody (antigoat) at 4 °C. Following incubation, lysates were incubated with Dynabeads Protein G (Invitrogen) (pre-equilibrated with lysis buffer) for 1 h at 4 °C. The Dynabeads were denatured using 2× Laemmli buffer containing 8% β-mercaptoethanol for 8 min at 80 °C.

Samples were separated on Bolt 4–12% Bis-Tris Plus precast gels (Life Technologies) and transferred onto the nitrocellulose membrane (GE Healthcare Life Sciences) using the semidry transfer Novex system (Life Technologies). The membranes blocked using Odyssey blocking buffer (Li-Cor) were incubated with primary antibodies (INTS3 antirabbit and hSSB1 antigoat) overnight at 4 °C in a 1:1 solution of Odyssey blocking buffer and PBS-T. All primary antibodies were used at a dilution of 1:1000. The membrane was then washed with PBS-T and incubated with secondary antibodies, donkey antirabbit, and donkey antigoat (at a dilution of 1:10000). The membrane was again washed with PBS-T and imaged by using the Li-Cor Odyssey system (Li-Cor).

#### Immunofluorescence and High Content Microscopy

U2OS cells (1.0 × 10^4^ cells/well) were seeded on a glass-base 96-well plate (Corning) and left to adhere for 8 h before compound treatment. For treatments, cells were exposed to three concentrations of each compound (0.5, 5, and 10 μM). After 24 h, the treated cells were washed with PBS and cultured in fresh media containing respective compound treatments for another 12 h. The cells were subjected to IR treatment at a dose of 6 Gy. Cells were fixed with 4% paraformaldehyde (PFA) in PBS for 20 min at room temperature (RT) and permeabilized with 0.1% Triton X-100 in PBS for 5 min at ambient temperature. The cells were subsequently blocked with 2% donkey serum in PBS for 30 min at ambient temperature. Primary antibodies were diluted in 0.5% donkey serum in PBS and incubated overnight at 4 °C. Antibodies targeting INTS3 and hSSB1 were used at dilutions of 1:300 and 1:700, respectively. Fluorescent secondary antibodies conjugated with the Alexa Fluor dye were diluted in 0.5% donkey serum in PBS and incubated for 1 h at RT (1:600 dilution). Following this, the cells were stained with DAPI stain diluted in PBS (final concentration of 1 μg/mL) and incubated at RT for 5 min before imaging. Images were collected on an InCell Analyzer 6500 high content microscopy imaging system (GE Healthcare Life Sciences) 6500 HS microscope equipped with IN Cell Analyzer 6500 v7.4 software. Images were analyzed using the Cell Profiler software v3.1.9. INTS3 and hSSb1 foci were reported as foci per nuclei per field of view, *n* = 25 fields.

### Analysis and Visualization

#### *InSilico* Studies

The trajectories of MD simulations were created and analyzed using the CPPTRAJ module of AmberTools18.^56^ The visualization of molecular docking poses, clustering of simulation trajectories, and preparation of MD movies and images were performed using UCSF Chimera v1.16^41^ and ChimeraX v1.5.^66,67^

#### *In Vitro* Studies

The western blots were imaged using an Odyssey system (Li-Cor). The analysis of immunofluorescence stains was carried out using CellProfiler v3.1.9 cell image analysis software. All the data analyses for plotting of figures were carried out using either GraphPad Prism v9.4.1 or Python3.

## Supporting Information Available

Figure S1: RMSF plot of INTS3 in its apo and holo state (when bound to compounds, I1-I5) (PDF)
