# Supplementary material for: Targeting the hSSB1-INTS3 Interface: A Computational Screening Driven Approach to Identify Potential Modulators
Source: ACS Omega. 2024 Feb 8;9(7):8362–73. doi: 10.1021/acsomega.3c09267 (PMC10882649; doi:10.1021/acsomega.3c09267)
Supplement: Supplementary file 1 — ao3c09267_si_001.pdf [file ao3c09267_si_001.pdf]

Targeting the hSSB1-INTS3 interface: A computational screening driven approach to identify potential modulators

Tabassum Khair Barbhuiya[1, 2], Sam Beard[2, 3], Esha T. Shah[2, 3,] Steven Mason[4], Emma Bolderson[2,3], Ken O'Byrne[2, 3], Luke W. Guddat[4], Derek J. Richard[2,3], Mark N. Adams[2,3*], Neha S. Gandhi[5(†),1,2*]

1. Centre for Genomics and Personalised Health, and School of Chemistry and Physics, Faculty of Science, Queensland University of Technology, 2 George Street, Brisbane, QLD 4000, Australia
2. Cancer and Ageing Research Program, Woolloongabba, QLD 4102, Australia
3. Centre for Genomics and Personalised Health, and School of Biomedical Sciences, Faculty of Health, Queensland University of Technology, Kelvin Grove QLD 4059, Australia
4. School of Chemistry and Molecular Biosciences, The University of Queensland, Brisbane QLD 4072, Australia
5. Department of Computer Science and Engineering, Manipal Institute of Technology, Manipal Academy of Higher Education, 576104, Manipal, INDIA (†).

* Corresponding authors

† Current Affiliation

Emails of Corresponding Authors:

Neha S. Gandhi: neha.gandhi@manipal.edu, neha.gandhi@qut.edu.au,

Mark N. Adams: mn.adams@qut.edu.au

Supporting Information

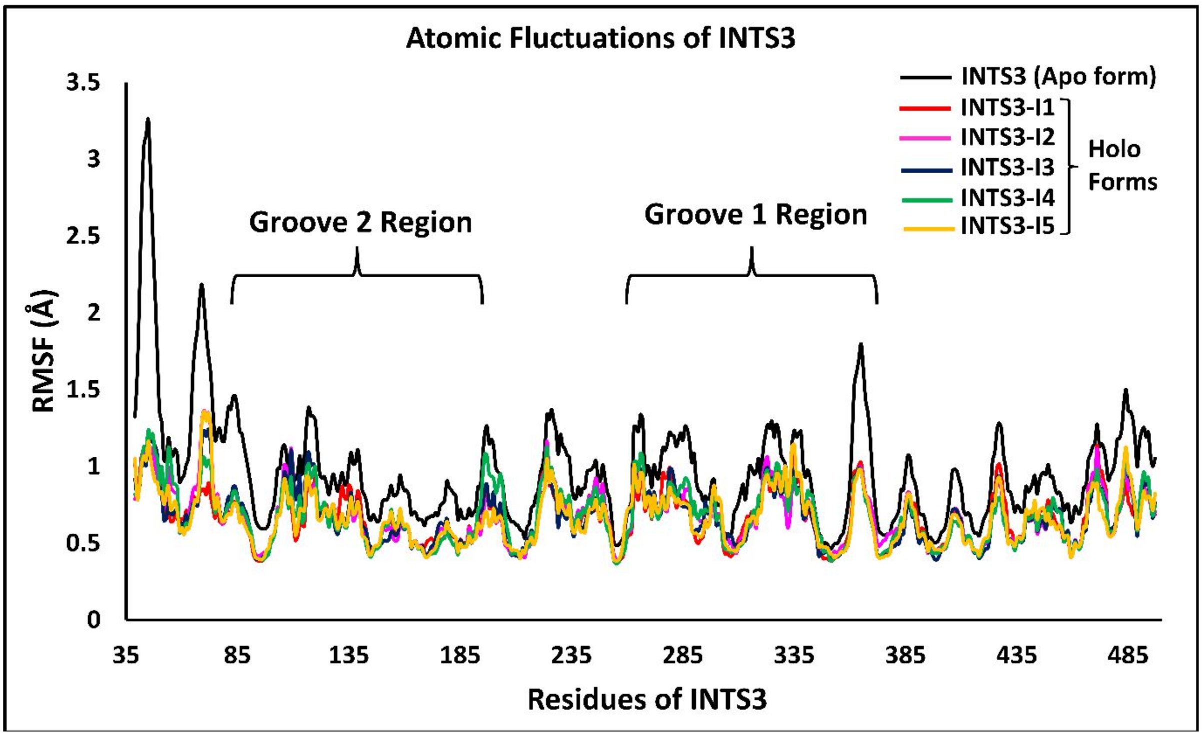

Figure S1: *Plot of root mean squared fluctuations of INTS3 atoms in its apo and holo states (when bound to compounds, I1-I5)*
